# Prepared for PrEP: preferences for HIV pre-exposure prophylaxis among Chinese men who have sex with men in an online national survey

**DOI:** 10.1186/s12879-019-4692-x

**Published:** 2019-12-16

**Authors:** Wenting Huang, Dan Wu, Jason J. Ong, M. Kumi Smith, Fan Yang, Hongyun Fu, Weiming Tang, Joseph D. Tucker

**Affiliations:** 1 University of North Carolina at Chapel Hill Project-China, Guangzhou, China; 2 Population Health Science, Bristol Medical School, University of Bristol, Bristol, UK; 3 London School of Hygiene and Tropical Medicine, London, UK; 4 Division of Epidemiology and Community Health, University of Minnesota Twin Cities, Twin Cities, USA; 5 Eastern Virginia Medical School, Norfolk, USA; 6 Dermatology Hospital, Southern Medical University, Guangzhou, China; 7 School of Medicine, University of North Carolina at Chapel Hill, Chapel Hill, NC 27599 USA

**Keywords:** HIV, Men who have sex with men, Pre-exposure prophylaxis, Preference, China

## Abstract

**Background:**

Pre-exposure prophylaxis (PrEP) is not widely available in China. Previous studies reported low awareness and inconclusive findings on the acceptability of PrEP among Chinese men who have sex with men (MSM).

**Methods:**

We conducted a secondary analysis of an online national survey comparing preferences for oral and long-acting injectable PrEP among MSM and identifying correlates of preferences. The study did not collect detailed information about partner types that may influence negotiated safety and PrEP uptake.

**Results:**

Nine-hundred and seventy-nine men from the larger sample of 1045 men responded to the PrEP survey questions. Most men (81.9%) had never heard of PrEP, but reported interest in using PrEP. More participants chose injectable PrEP (36.3%) as their preferred formulation than oral PrEP (24.6%). Men who had at least two HIV tests (adjusted OR = 1.36, 95%CI 1.04, 1.78) more commonly preferred injectable PrEP.

**Conclusion:**

Our findings may help inform PrEP messaging in areas where PrEP has yet to be scaled up.

## Background

Pre-exposure prophylaxis (PrEP), one of the most effective biological tools to prevent HIV acquisition, has been recommended by the World Health Organization (WHO) for individuals with increased risk of HIV infection, including some men who have sex with men (MSM) [1]. Several trials have demonstrated the effectiveness of PrEP among high-risk MSM [2, 3]. PrEP uptake has increased in some countries over the past several years, but rates are still modest in most countries [4]. Expanding PrEP uptake in countries could avert many HIV infections. For example, in China, a modeling study indicated that a PrEP coverage of 50% in high-risk MSM could prevent 170,000 to 320,000 of new HIV infections over the next two decades [5]. However, PrEP is not yet approved for clinical use in China.

PrEP uptake barriers among MSM include low awareness, high cost, limited support from partners, low perceived HIV risk, limited access, and potential problems with adherence [6–12]. In addition, cultural beliefs about health, HIV stigma, homophobia, and limited health insurance coverage for PrEP are further barriers to oral PrEP uptake [13]. To overcome some of these barriers, alternative formulations like long-acting injectables have shown promise in several clinical trials [14, 15]. Long-acting injectable PrEP could potentially simplify HIV prevention services, reduce HIV stigma associated with pill taking, and improve HIV prevention services [16]. A national study in the US found that among gay and bisexual men who were willing to use PrEP, men preferred long-acting injectable PrEP compared to oral forms [17].

However, less is known about preferences for injectable PrEP in China and other low- and middle-income countries [18]. Some studies suggest that MSM in China have low awareness of PrEP (11.2–33.5%), but high willingness (54.0–67.8%) to potentially use daily PrEP [8, 16, 19]. Chinese MSM sometimes leave the country in order to purchase PrEP [20]. Conversely, studies in Shanghai and Hong Kong reported low willingness and low actual uptake of oral PrEP [18, 19]. The Shanghai study also found poor adherence among those who initiated PrEP [18]. More information about PrEP preferences among MSM would be useful for the introduction and scale-up of PrEP in China and other locations where PrEP is not widely available. The purpose of this study was to compare preferences for oral and long-acting injectable PrEP among MSM and identify correlates of preferences.

## Methods

### Participants and procedures

This is a secondary analysis of data collected from a cross-sectional online national study on HIV testing preferences among Chinese MSM [21]. Participants were recruited from January 8 to January 312,017 through banner advertisements on web- and mobile app-based platforms on Blued, a large gay Chinese social media platform. Eligible participants completed an online survey. Eligibility criteria included the following: 1) born biologically male; 2) older than age 16 years; 3) had anal or oral sex with another man; 4) not diagnosed with HIV. In order to address some of the shortcomings of online sampling, we assigned sample quotas for each of the following subgroups: annual income level above and below 5500 USD, educational level above and below high school, and have and have not disclosed MSM behaviors to a medical provider. Detailed recruitment and sampling strategies can be found in a previous paper [21]. Participants received $7.50 USD mobile data credits as compensation for their time to complete the 400-item survey, which took approximately 30 min to finish. This analysis only included men who responded to at least one survey question on PrEP .

### Measures

#### Socio-demographics

Socio-demographic characteristics were extracted from the dataset including age (continuous), gender identity (male, female, transgender, or unsure/other), sexual orientation (gay, bisexual, or other), educational level (high school or below versus above high school), annual income (USD 5500 or below versus above USD 5500), and marital status (never married versus ever married). Disclosure of one’s sexual orientation to medical provider was also extracted .

#### Factors related to sexual risk behavior and HIV testing behavior

Items assessing sexual risk behaviors included seeking sexual partners online in the last 12 months (yes/no), number of male sexual partners in the last 3 months (individuals who have more than one male partner were classified as having multiple male sexual partners), condomless sex in the last 3 months (yes/no), and anal sex role (insertive, receptive, or both). We also asked participants whether they had tested for HIV and the number of HIV tests in the past year. Individuals who had more than one test were classified as have multiple HIV tests in the past year.

#### Community engagement in sexual health

In this study, community engagement in sexual health was defined as awareness, participation, and advocacy for sexual health in the community. Community engagement was measured by a six-item scale validated among Chinese MSM [22]. The scale included the following items: discussed HIV/STI testing or sexual health online, aware of any MSM sexual health events, encouraged others to get HIV/STI tested, accompanied others to a HIV/STI testing facility, helped organize a MSM sexual health campaign, and volunteered to help provide MSM sexual health services [22]. These community engagement items were categorized into none, minimal, moderate, and substantial engagement according to previous studies [22].

#### PrEP acceptability and formulation preferences

Interest in using PrEP and preferences regarding PrEP formulation were adapted from a study in Vietnam [23] which included three formulations: oral PrEP, long-acting injectable PrEP, and rectal microbicides.

Before completing the survey, participants were provided with a brief introduction to PrEP (Additional file 1: Table S1). PrEP was described an evidence-based approach to prevent HIV infection among people at high risk of infection. Oral PrEP was described as a daily pill. The effectiveness of oral PrEP was given as reducing 92% of the risk of HIV infection when taken consistently. Long-acting injectable PrEP was described as an injection or shot given every 3 months and the effectiveness was described as unclear. Rectal microbicide was described as a gel (like a lubricant) inserted into the rectum before sex and the effectiveness was described as unclear. In this study, we focus on the correlates with interests and preferences on oral and long-acting injectable PrEP.

Participants were asked whether they had heard about PrEP before this survey. Participants were then asked how interested they would be in taking each PrEP formulation if it were made available in China. Their answers were recoded as interested (very interested, somewhat interested, interested) and not interested (neutral, somewhat uninterested, very uninterested, not sure) in using PrEP. PreP formulation preferences were asked as follows: “Please rank your preferred PrEP formulation (from the most to the least preferred)”. Participants who had no interest in PrEP were able to skip these questions. Participants who ranked injectable PrEP as their most preferred formulation were defined as preference for injectable PrEP.

### Statistical analysis

The sample for this analysis included 979 MSM. The sample excluded men who did not answer PrEP questions (*n* = 66). Descriptive analyses were used to summarize socio-demographic characteristics and sexual behaviors of the sample. Logistic regression models were used to identify correlates of the following: interest in using oral PrEP; interest in using injectable PrEP; preference for injectable PrEP compared to other formulations. Bivariate logistic regression models were used to test the association between PrEP outcomes with disclosure to medical provider, PrEP awareness, sexual risk behavior, HIV testing behaviors, and community engagement in sexual health. These variables were chosen based on previous published literature in China and factors that have influenced PrEP uptake in other settings. We examined associations between sexual risk behaviors and PrEP preferences, adjusting for age, education, and annual income. Odds ratios (OR) and adjusted odds ratios (aOR) were reported with 95% confidence intervals (95%CI) in logistic regression models. An alpha level of 0.05 or less was considered statistically significant. All statistical analyses were conducted using Stata/SE 13.0 (StataCorp, Texas, USA).

## Results

Men who did not respond to the PrEP question (*n* = 66) were similar to men who responded to this question (Additional file 1: Table S2). Among the 979 MSM in this secondary analysis, the median age was 26 (ranged 16–59) (Table 1). They mostly self-identified as gay (768, 78.5%) and most were unmarried (835, 85.3%). More than three-quarters of the participants had sought a sexual partner online in the last 12 months (745, 76.1%). About a third of participants (358, 36.6%) had multiple sexual partners, and around half (499, 51.0%) had condomless sex in the last 3 months. Most men had previously tested for HIV (697/979, 71.2%), but only 42.9% (299/697) had at least two HIV tests in the last year. Fewer than half of men (44.9%, *n* = 426) had a moderate level of community engagement in sexual health and 31.8% (*n* = 302) of men had substantial engagement.

**Table 1:** Characteristics of men who have sex with men in China, 2017 (*N* = 979)

	*n* (%)
Age (years)	
16–24	487 (49.7)
> 24	492 (50.3)
Gender identity	
Men	908 (92.8)
Women	24 (2.5)
Transgender	14 (1.4)
Unsure/other	33 (3.4)
Sexual orientation	
Gay	768 (78.5)
Bisexual	172 (17.6)
others	39 (4.0)
Educational level	
High school or below	425 (43.4)
Above high school	554 (56.6)
Annual income, US$	
5500 or below	482 (49.2)
Above 5500	497 (50.8)
Marital status with women	
Never married	835 (85.3)
Ever married	144 (14.7)
Disclosed sexual orientation to medical provider	
No	520 (53.1)
Yes	459 (46.9)
Partner seeking online^a^	
No	234 (23.9)
Yes	745 (76.1)
Multiple male sexual partners^b^	
No	621 (63.4)
Yes	358 (36.6)
Condomless sex^b^	
No	480 (49.0)
Yes	499 (51.0)
Role during anal sex^b^	
Both	568 (54.4)
Insertive	188 (18.0)
Receptive	240 (23.0)
Multiple HIV tests^a,c^	
No	581 (59.4)
Yes	398 (40.7)
Community Engagement^d^	
No	121 (12.4)
Minimal	101 (10.3)
Moderate	426 (43.5)
Substantial	302 (30.9)

^a^in the last 12 months

^b^in the last 3 months

^c^two or more HIV tests

^d^*N* = 950

Overall, 81.9% (802/979) of the participants had never heard of PrEP. Following a brief introduction about PrEP, 85.7% (836/979) of men were interest in using long-acting injectable PrEP and 76.7% (751/979) of men were interested in using oral PrEP (Table 2). Among the 751 participants who were interested in using oral PrEP, 93.9% (705/751) also reported interest in using injectable PrEP. Of the remaining 23.3% (228/979) MSM who were not interested in using oral PrEP, 57.5% (131/228) were interested in using injectable PrEP. 90% (882/979) of men were interested in using at least one formulation of PrEP. Almost all participants (98.6%, 965/979) completed the item that asked them to rank PrEP formulations in order of preference. 39.2% (*n* = 378) of men ranked rectal microbicides as their most preferred formulation and 36.3% (*n* = 350) of men ranked injectable PrEP as preferred. 24.6% (*n* = 237) of men ranked oral PrEP as their most preferred formulation. There was no significant difference in PrEP formulation preference between participants who who had higher risk sexual behaviors (those who had either multiple sexual partners or had condomless sex in the last 3 months) and the overall sample.

**Table 2:** Awareness and preferences for PrEP formulation in Chinese MSM, 2017 (*N* = 979)

	*n*	%	95%CI^c^
Ever heard about PrEP			
No	802	81.9	79.5, 84.3
Yes	177	18.1	15.7, 20.5
Interested in using oral PrEP			
No or not sure	228	23.3	20.7, 26.0
Yes	751	76.7	74.0, 79.3
Interested in using injectable PrEP ^a^			
No or not sure	140	14.3	12.3, 16.7
Yes	836	85.7	83.3, 87.7
Preference of PrEP formulations ^b^			
Oral PrEP	237	24.6	21.9, 27.4
Injectable PrEP	350	36.3	33.3, 39.4
Rectal Microbicide	378	39.2	36.1, 42.3

^a^3 missing values

^b^Participants were categorized by their first ranked PrEP formulation preference

^c^Confidence interval

Table 3 presents the associations between sexual risk behaviors and interest in PrEP formulations. Adjusting for the socio-demographic variables (age, education, and income), men who had ever heard about PrEP (aOR = 1.57, 95%CI 1.02, 2.41), men who had sought sexual partners online in the last 12 months (aOR = 1.64, 95%CI 1.18, 2.29), men who had multiple male sexual partners in the last 3 months (aOR = 1.81, 95%CI 1.30, 2.52), and men who had a higher level of community engagement in sexual health (aOR =2.67, 95%CI 1.68, 4.27) were more likely to be interested in oral PrEP. Men who had sought sexual partners online (aOR = 2.10, 95%CI 1.43, 3.08), men who had multiple sexual partners (aOR = 1.76, 95%CI 1.18, 2.63), men who had multiple HIV tests in the past year (aOR = 1.94, 95%CI 1.31, 2.88), and men who had a higher level of community engagement in sexual health (aOR = 2.25, 95%CI 1.34, 3.79) were more likely to be interested in injectable PrEP.

**Table 3:** Factors and risk behaviors associated with interests in using oral PrEP (*N* = 979) and injectable PrEP (*N* = 976) compared to those are not interested among Chinese MSM, 2017

	Oral PrEP		Injectable PrEP	
	OR (95%CI^f^)	aOR^a^ (95%CI^f^)	OR (95%CI^f^)	aOR^a^ (95%CI^f^)
Disclosed to medical provider				
No	Ref	Ref	Ref	Ref
Yes	1.12 (0.83, 1.51)	1.22 (0.90, 1.65)	1.10 (0.77, 1.58)	1.19 (0.83, 1.72)
Ever heard about PrEP				
No	Ref	Ref	Ref	Ref
Yes	1.61 (1.05, 2.45)*	1.57 (1.02, 2.41)*	1.30 (0.79, 2.13)	1.26 (0.76, 2.08)
Partner seeking online^b^				
No	Ref	Ref	Ref	Ref
Yes	1.68 (1.21, 2.33)*	1.64 (1.18, 2.29)*	2.12 (1.45, 3.11)*	2.10 (1.43, 3.08)*
Multiple male sexual partners^c^				
No	Ref	Ref	Ref	Ref
Yes	1.69 (1.22, 2.35)*	1.81 (1.30, 2.52)*	1.66 (1.11, 2.47)*	1.76 (1.18, 2.63)*
Condomless sex^c^				
No	Ref	Ref	Ref	Ref
Yes	1.21 (0.90, 1.62)	1.23 (0.91, 1.66)	0.98 (0.69, 1.40)	0.99 (0.69, 1.43)
Role during anal sex^c^				
Both	Ref	Ref	Ref	Ref
Insertive	0.74 (0.51, 1.08)	0.75 (0.51, 1.10)	0.63 (0.40, 0.98)*	0.64 (0.40, 1.00)
Receptive	0.89 (0.62, 1.27)	0.81 (0.56, 1.17)	0.72 (0.47, 1.10)	0.66 (0.42, 1.02)
Multiple HIV tests^b, d^				
No	Ref	Ref	Ref	Ref
Yes	1.39 (1.02, 1.90)*	1.45 (1.06, 1.98)*	1.86 (1.26, 2.76)*	1.94 (1.31, 2.88)*
Community engagement in sexual health^e^				
No	Ref	Ref	Ref	Ref
Minimal	2.28 (1.25, 4.14)*	2.18 (1.19, 3.98)*	3.27 (1.52, 7.05)*	3.16 (1.46, 6.83)*
Moderate	2.21 (1.44, 4.14)*	2.20 (1.42, 3.41)*	2.77 (1.67, 4.57)*	2.77 (1.67, 4.60)*
Substantial	2.56 (1.61, 4.07)*	2.67 (1.68, 4.27)*	2.17 (1.29, 3.63)*	2.25 (1.34, 3.79)*

Logistic regressions use people who were not interested in using oral/injectable PrEP as reference level, do not include people who reported not sure or do not know whether they were interested in using oral or injectable PrEP. Bold refers to associations that have a 95% CI that does not include 1

^a^Adjusted variables included age, education, and annual income

^b^in the last 12 months

^c^in the last 3 months

^d^two or more HIV tests

^e^*N* = 950

^f^Confidence interval

*p < 0.05

Table 4 presents the associations between sexual risk behaviors and a preference for injectable PrEP using bivariate and multivariable logistic regression. We adjusted for age, education, and income in multivariable logistic regression. Participants who had ever heard about PrEP (aOR = 1.55, 95%CI 1.11, 2.17) and who had multiple HIV tests in the last year (aOR = 1.36, 95%CI 1.04, 1.78) were more likely to rank injectable PrEP as their most preferred PrEP formulation. Men who only had recent receptive anal sex were more likely to prefer injectable PrEP compared to men who had both recent receptive and insertive anal sex. (aOR = 0.61, 95%CI 0.43, 0.85).

**Table 4:** Characteristics associated with preferred PrEP formulation versus the rest formulations among Chinese MSM, 2017 (*N* = 965)

	Injectable PrEP	
	OR (95%CI^f^)	aOR^a^ (95%CI^f^)
Disclosed to medical provider		
No	Ref	Ref
Yes	1.26 (0.99, 1.67)	1.26 (0.96, 1.64)
Ever heard about PrEP		
No	Ref	Ref
Yes	1.54 (1.10, 2.14)*	1.55 (1.11, 2.17)*
Partner seeking online^b^		
No	Ref	Ref
Yes	0.86 (0.63, 1.16)	0.86 (0.63, 1.17)
Multiple sexual partners^c^		
No	Ref	Ref
Yes	1.04 (0.80, 1.37)	1.02 (0.78, 1.35)
Condomless sex^c^		
No	Ref	Ref
Yes	0.81 (0.62, 1.05)	0.81 (0.62, 1.06)
Role during anal sex^c^		
Both	Ref	Ref
Insertive	0.84 (0.59, 1.19)	0.82 (0.58, 1.17)
Receptive	0.60 (0.43, 0.84)*	0.61 (0.43, 0.85)*
Multiple HIV tests^b, d^		
No	Ref	Ref
Yes	1.38 (1.05, 1.80)*	1.36 (1.04, 1.78)*
Community engagement in sexual health^e^		
No	Ref	Ref
Minimal	1.43 (0.82, 2.49)	1.46 (0.83, 2.55)
Moderate	1.21 (0.78, 1.87)	1.20 (0.78, 1.87)
Substantial	1.48 (0.94, 2.32)	1.46 (0.93, 2.29)

Other PrEP formulations include oral PrEP and rectal microbicides. Bold refers to associations that have a 95% CI that does not include 1

Logistic regressions adjusted variables include age, education, and annual income

^a^Adjusted variables included age, education, and annual income

^b^in the last 12 months

^c^in the last 3 months

^d^two or more HIV tests

^e^*N* = 950

^f^Confidence interval

*p < 0.05

## Discussion

This study contributes to the literature on PrEP acceptability by examining preferences for oral and long-acting injectable PrEP using an online sample of Chinese MSM. In this study, we found low awareness of PrEP, but a high interest in using oral and injectable PrEP. We also found that long-acting PrEP could be an alternative formulation of PrEP that would be preferred by large numbers of Chinese MSM. This study expands the literature on PrEP in China by including a nationwide sample of MSM that purposefully over-representing low-income and closeted MSM who are difficult to reach using conventional sampling methods.

We found that many MSM in China were unaware of PrEP. This finding is consistent with earlier studies assessing PrEP awareness rates in China [7, 8, 11, 12]. The awareness rates we observed in this study are much lower than rates reported in the US, Canada, and Brazil [24–26]. Low PrEP awareness among MSM in China may have been related to the lack of an approved drug, lack of Chinese guidelines, lack of support for MSM communities, and limited drug availability [8, 13]. This finding suggests the need for further community engagement to increase PrEP awareness among Chinese MSM.

We found that PrEP interest was associated with receiving more than one HIV test in a year. MSM who received more than one HIV test in a year may either have more risk behaviors or higher self-perceived risk of acquiring HIV [11, 16, 27]. This finding also suggests that PrEP services need to be integrated with HIV testing services. Many countries have used HIV testing services as an entry point for PrEP delivery, adherence monitoring, and follow-up [3, 28, 29]. The expansion of fourth-generation HIV testing in many Chinese cities would help detect early HIV infections and provide an opportunity for PrEP enrollment.

Our data suggest that many Chinese MSM prefer injectable PrEP compared to oral PrEP. This finding is consistent with data from China [16] and the US [30]. The preference for injectable PrEP may be due to convenience, decreased frequency of medication taking, and less stigma [30, 31]. Compared with taking daily oral PrEP, long-acting injectable PrEP may be more discreet and decrease the risk of being outed as gay [32]. Injectable PrEP may be preferred by Chinese MSM who have not yet disclosed their sexual orientations to others because of the fear of HIV stigma and homophobia. Furthermore, long-acting injectable PrEP could simplify adherence monitoring.

Our study has several limitations. First, the PrEP interest was self-reported rather than ascertained by uptake and most men had not heard of PrEP before this study. Although some early studies in China suggested a low uptake of PrEP among MSM, these pilot studies were done with minimal community engagement [33, 34]. Our finding of high interest in using PrEP indicates that a considerable proportion of Chinese MSM may be interested in receiving further information if they have an introduction. However, considering the complexity of PrEP usage, future research could focus more messaging related to PrEP use, side effects, adherence, and financial support. We also did not ask participants about specific forms of negotiated safe sexual partnerships which may influence sexual risk taking and PrEP interest. There may be unplanned or unspoken safe arrangements between MSM that influence PrEP interest. Second, the PrEP formulation preference was based on three options and associated descriptions provided in the survey. Caution needs to be taken when generalizing this finding to MSM who are more familiar with PrEP. At the same time, this finding may be relevant in many locations where PrEP is not widely available. Third, the measure of community engagement in sexual health was focused on health services. To better measure men’s engagement with the gay community, qualitative methods or more community-focused scales would be useful. Fourth, selection bias may have been introduced by the online data collection approach. However, the quota we assigned during the recruitment ensured MSM with lower education, lower income, and less disclosure. Finally, we did not explore the participants’ willingness to pay for PrEP. But given the early stage of adopting PrEP in China, findings of this study are still valuable in providing evidence for future PrEP trials and formative programs.

## Conclusions

Most Chinese MSM were unaware of PrEP but were willing to use it once they learned about it. Future studies should consider integrating biomedical prevention with behavioral intervention and HIV testing services. Involving MSM communities to promote long-acting injectable formulation of PrEP with oral PrEP may be more effective than promoting only oral PrEP through health facilities.

## Supplementary information

**Additional file 1: Table S1.** Descriptions of PrEP formulations. **Table S2.** Characteristics of participants who did not answer PrEP related question in China, 2017 (*N* = 66).

## Data Availability

The datasets used and/or analyzed during the current study are available from the corresponding author on reasonable request.

## Abbreviations

CI: Confidence interval
MSM: Men who have sex with men
OR: Odds ratio
PrEP: Pre-exposure prophylaxis
