# Correction to: Prepared for PrEP: preferences for HIV pre-exposure prophylaxis among Chinese men who have sex with men in an online national survey

**DOI:** 10.1186/s12879-020-4906-2

**Published:** 2020-02-27

**Authors:** Wenting Huang, Dan Wu, Jason J. Ong, M. Kumi Smith, Fan Yang, Hongyun Fu, Weiming Tang, Stephen Pan, Joseph D. Tucker

**Affiliations:** 1 University of North Carolina at Chapel Hill Project-China, Guangzhou, China; 2 Population Health Science, Bristol Medical School, University of Bristol, Bristol, UK; 3 London School of Hygiene and Tropical Medicine, London, UK; 4 Division of Epidemiology and Community Health, University of Minnesota Twin Cities, Twin Cities, USA; 5 Eastern Virginia Medical School, Norfolk, USA; 6 Dermatology Hospital, Southern Medical University, Guangzhou, China; 7 Xi’an Jiaotong-Liverpool University, Wuzhong District, Suzhou, China; 8 School of Medicine, University of North Carolina at Chapel Hill, Chapel Hill, NC 27599 USA

**Correction to: BMC Infect Dis (2019) 19:1057**

**https://doi.org/10.1186/s12879-019-4692-x**

After publication of the original article [1], the authors would like to add a co-author, Dr. Stephen Pan, who contributed sufficiently to this manuscript. He was not included in the original article due to a miscommunication.
