# Supplementary material for: Prepared for PrEP: preferences for HIV pre-exposure prophylaxis among Chinese men who have sex with men in an online national survey
Source: BMC Infect Dis. 2019 Dec 16;19:1057. doi: 10.1186/s12879-019-4692-x (PMC6916236; doi:10.1186/s12879-019-4692-x)
Supplement: Supplementary file 1 — Additional file 1: Table S1. Descriptions of PrEP formulations. Table S2. Characteristics of participants who did not answer PrEP related question in China, 2017 (N = 66). [file 12879_2019_4692_MOESM1_ESM.docx]

Table S1. Descriptions of PrEP Formulations

| PrEP formulation | Descriptions |
| --- | --- |
| Oral PrEP | PrEP (pre-exposure prophylaxis) is a new biochemical strategy to prevent HIV infection. PrEP involves HIV-negative guys taking anti-HIV medications (for example, Truvada) once a day, every day to reduce the likelihood of HIV infection if they were exposed to the virus through sex or injecting drug use. Clinical trials of PrEP indicated that it reduced92% of the likelihood of HIV infection when used consistently. |
| Long-acting injectable PrEP | Another type of PrEP that would involve getting an injection every three months is under studying. Scientists are comparing its effectiveness in protecting HIV infection with daily oral PrEP. |
| Rectal microbicides | Another type of PrEP, rectal microbicide, were also under studying, which requires being injected into rectum before sex. Scientists are comparing its effectiveness in protecting HIV infection with daily oral PrEP. |

Table S2. Characteristics of participants who did not answer PrEP related question in China, 2017 (*N*=66)

|  |  | *n* (%) |
| --- | --- | --- |
| Age (years) | 16-24 | 31 (47.0) |
|  | >24 | 35 (53.0) |
| Gender identity | Men | 57 (86.4) |
|  | Women | 5 (7.6) |
|  | Transgender | 2 (3.0) |
|  | Unsure/other | 2 (3.0) |
| Sexual orientation | Gay | 51 (77.3) |
|  | Bisexual | 11 (16.7) |
|  | others | 4 (6.1) |
| Educational level | High school or below | 24 (36.4) |
|  | Above high school | 42 (63.6) |
| Annual income, US$ | 5500 or below | 28 (42.4) |
|  | Above 5500 | 38 (57.6) |
| Marital status with women^1^ | Never married | 23 (74.2) |
|  | Ever married | 8 (25.8) |
| Disclosed sexual orientation to medical provider | No | 28 (42.4) |
|  | Yes | 38 (57.6) |
| Partner seeking online^2^ | No | 55 (83.3) |
|  | Yes | 11 (16.7) |
| Multiple male sexual partners^3,4^ | No | 11 (63.4) |
|  | Yes | 6 (36.6) |
| Condomless sex^3,4^ | No | 8 (47.1) |
|  | Yes | 9 (52.9) |
| Role during anal sex^3,4^ | Both | 11 (64.7) |
|  | Insertive | 1 (5.9) |
|  | Receptive | 5 (29.4) |
| Ever been tested for HIV^5^ | No | 3 (18.8) |
|  | Yes | 13 (81.3) |
| Multiple HIV tests^2,5^ | No | 64 (97.0) |
|  | Yes | 2 (3.03) |
| Community Engagement^¶^ | No | 0 (0) |
|  | Minimal | 0 (0) |
|  | Moderate | 0 (0) |
|  | Substantial | 0 (0) |

^1^ *N*=31

^2^ in the last 12 months

^3^ in the last 3 months

^4^ *N*=17

^5^ two or more HIV tests

^6^ *N*=16

^¶^ no observations
